# Traumatic submacular hemorrhage: available treatment options and synthesis of the literature

**DOI:** 10.1186/s40942-019-0200-0

**Published:** 2019-12-11

**Authors:** Giamberto Casini, Pasquale Loiudice, Martina Menchini, Francesco Sartini, Stefano De Cillà, Michele Figus, Marco Nardi

**Affiliations:** 1 Ophthalmology Unit, Department of Surgical, Medical, Molecular and Critical Area Pathology, University of Pisa, Pisa, Italy; 2 Department of Health Sciences, Eye Clinic, University of Piemonte Orientale, Novara, Italy

**Keywords:** Anti-vascular endothelial grow factor, Blunt ocular trauma, Pneumatic displacement, Vitrectomy, Submacular hemorrhage, Tissue plasminogen activator

## Abstract

Sub-macular hemorrhage (SMH) is a hematic collection between the neurosensory retina and the retinal pigment epithelium; one of its causes is ocular blunt trauma, that usually affects young patients. Persisting SMH leads to a damage of photoreceptors mediated by three main mechanisms: iron-related toxicity, impairment of diffusion of oxygen and nutriment, mechanical damage due to clot contraction. Since early photoreceptors’ damage has been reported within 24 h, it is suggested to provide an early treatment, although there are no guidelines or consensus between authors regarding treatment strategies. The aim of this review was to present and compare available treatment options, like intravitreal tissue plasminogen activator (tPA) associated with pneumatic displacement, pneumatic displacement alone, subretinal tPA injection with pneumatic displacement, and intravitreal anti-vascular endothelial growth factor (VEGF) injection. All procedures obtained consistent results, though the most effective seemed to be pars plana vitrectomy, subretinal tPA and gas tamponade, probably due to a quicker liquefaction and displacement of the clot. Limitations concern the greater invasiveness and the higher incidence of complications. Alternatively, intravitreal injection of tPA and gas may represent a less invasive option with fewer complications. Intravitreal injection of gas and prone position could be preferred in young patients without coexisting ocular pathology, being a minimally invasive treatment, with lower risk of complications and a good visual recovery. Anti-VEGF agent have found, to date, limited employment in cases of traumatic SMH even though they may be useful as alternative or adjuvant therapy. Most of the published literature consists of small studies and case reports, therefore further investigations and larger clinical trials are required to fully understand safety and efficacy of the procedures. A preoperative comprehensive evaluation may be helpful to realize a surgical plan tailored on patient.

## Introduction

**Fig. 1:**
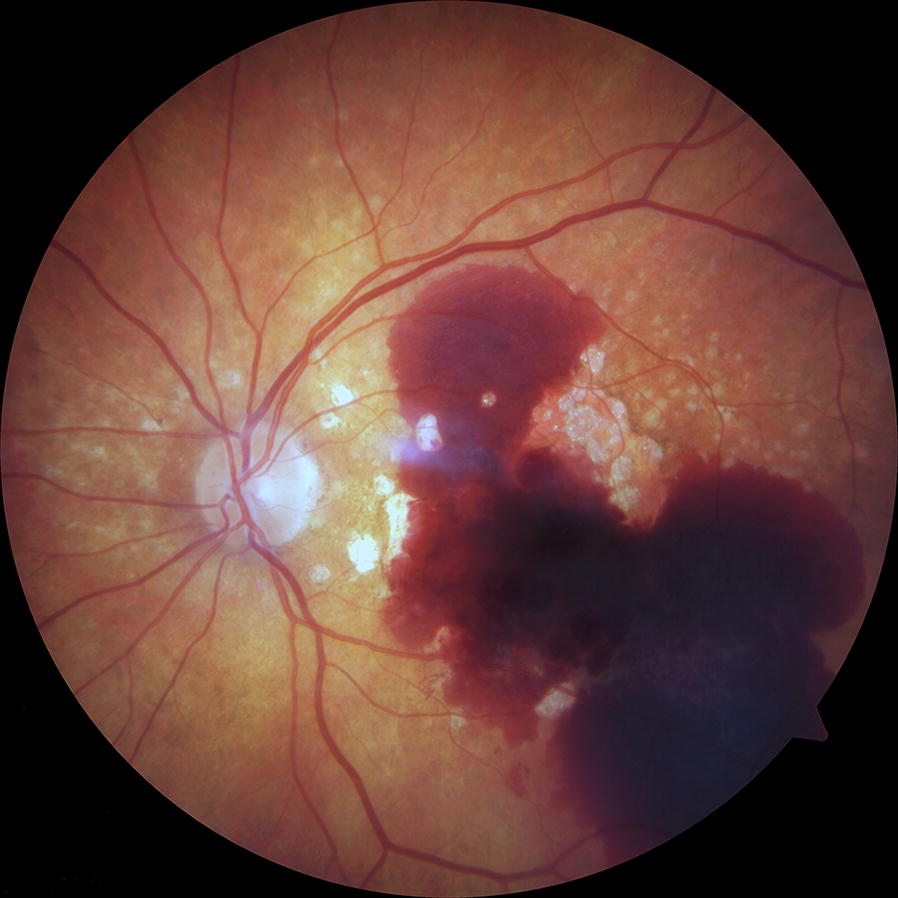
Fundus photograph of a patient with large submacular hemorrhage extending beyond the temporal vascular arcades

Sub-macular hemorrhage (SMH) is defined as blood collection between the neurosensory retina and the retinal pigment epithelium (RPE) (Fig. 1). It can be caused by several conditions, including age-related macular degeneration, pathologic myopia, polypoidal choroidal vasculopathy, macroaneurysms, presumed ocular histoplasmosis syndrome, and blunt trauma [1–5].

In pediatric age, the incidence of eye trauma has been reported to be 8.9–15.2/100 000 per year and about 62–65% of hospitalized trauma patients have blunt ocular trauma compared to 16–28% with an open-globe injury [6, 7]. Blunt ocular trauma, usually affecting young patients during sport activity or after episodes of violence, may lead to several pathologic consequences such as commotio retinae, choroidal rupture, macular hole, subretinal hemorrhage, retinal vascular occlusion, vitreous hemorrhage, retinal pigment epithelial edema, retinal dialysis, macular infarction, traumatic optic neuropathy and optic nerve avulsion [8–13].

In case of choroid rupture, the fracture usually runs parallel and temporal to the optic disk, with macula involvement in 66% of cases [14]. SMH, subsequent to choroidal rupture in macular region, is a sight-threatening condition, and is associated with poor visual outcome, especially if SMH persists for several days [15].

SMH may be classified according to the size: a small SMH measures less than 4 disc diameters, a medium-size SMH has a diameter greater than 4 disc diameters but does not extend beyond the temporal vascular arcade, a massive SMH overspreads the temporal vascular arcade [16].

Prompt treatment of SMH is recommended, even though there are no guidelines or consensus between authors regarding treatment strategies.

## Pathogenesis

Most of information regarding the pathogenesis of the damage SMH-related derived from animal models. Early hematic toxic effect can be demonstrated already 1 h after SMH onset in experimental rabbit models, consisting in edematous appearance of some photoreceptors [17]. Proceeding from day 1 to day 7, progressive damage was observed. Photoreceptor aspect ranged from edematous to absent, with pyknosis and karyolysis of the outer nuclear layer. At 2 weeks most of the clot was reabsorbed; photoreceptors and outer nuclear layer were completely absent; karyolysis of the inner nuclear layer was registered [17].

Macular damage due to SMH consists of a combination of multiple mechanisms. Blood coagulation produces erythrocyte degeneration, with release of iron and hemosiderin, and subsequent oxidative stress. Labile iron is one of the key factors for oxidative damage to cellular proteins, lipids, nucleic acids, and other cellular constituents [18]. Iron-mediated toxicity causes selective destruction of the first retinal neuron and has effects on retinal circulation [19]. Blood clot behaves like a barrier to metabolic exchange and limits the passage of nutrient and oxygen from the RPE to the photoreceptors. Since the metabolism of the photoreceptor is strongly related to RPE cell activity, this may explain because the former is more susceptible to the barrier effect [20]. Chemotaxis of macrophages and fibroblasts leads to release of inflammatory mediators and fibrin, leading to scar formation. Moreover, the clot in subretinal space adheres to photoreceptor; due to clot’s contraction, an avulsion of photoreceptor may occur [17, 20–24]. For this reason caution is suggest when a surgeon attempt to directly remove the clot from subretinal space.

## Prognostic factors

The natural history of SMH is poor, with final visual acuity ranging from 20/200 to light perception [25]. Visual prognosis depends on baseline visual acuity (VA), hemorrhage’s size and location, interval between SMH onset and treatment, concomitant involvement of anterior or posterior segment (e.g. iridodialysis, lens dislocation, traumatic cataract, vitreous hemorrhage and ciliary body clefts) [26–28].

Poor visual acuity at SMH onset, greater than 1.00 logMAR, is associated with poor functional outcome at 6 months [29]. Persistent SMH (more than 2 months) is related with worse final visual acuity [29]. The location and the extension of the hemorrhage are related with the functional recovery, especially in cases of massive SMH extending beyond the vascular arcades [30]. Contrarily, small size hemorrhage and younger age are predictor factors for better outcome [31]. Concomitant macular disease like polypoidal choroidal vasculopathy and age-related macular degeneration may influence the functional prognosis, being the former condition more inclined to recurrent hemorrhages, and the latter to the development of subretinal fibrosis [32–34].

Optical coherence tomography (OCT) can provide supplementary prognostic factors, such as retinal inner segment/outer segment layer integrity, foveal thickness and SMH area [35]. Intraoperative OCT can be used to identify the ideal location for needle penetration, and to grade the impact of surgical maneuvers on the retinal architecture [36]. During follow-up, OCT can be performed to estimate residual sub-macular blood and to assess the integrity of the underlying RPE in the choroidal rupture’s setting [28, 36–38].

## Therapeutic options

Management of traumatic SMH evolved over time and different approaches have been attempted, like intravitreal or subretinal injection of recombinant tissue plasminogen activator (tPA), intravitreal gas injection, a combination of them or intravitreal injections of anti-vascular endothelial growth factor (VEGF) agents (Table 1).

**Table 1:** Clinical data and treatment results in patients with traumatic sub macular hemorrhage

Author	No. of eyes	Treatment	Dose	Baseline BCVA	Final BCVA	Follow-up (months)
Balughatta et al. [22]	3	Intravitreous C3F8	0.3 ml	CF	20/30	3
				CF	20/40	
				20/60	20/90	
Ohji et al. [55]	5	Intravitreous C3F8	0.4 ml	20/400	20/15	14
			0.4 ml	20/300	20/40	6
			0.5 ml	20/2000	20/200	13
			0.4 ml	20/400	20/50	3
			0.4 ml	20/700	20/200	3
Gopalakrishan et al. [54]	4	Intravitreal C3F8	0.3 ml	CF	20/20	3
				CF	20/63	
				20/125	20/125	
				CF	20/20	
Tsuyama et al. [48]	1	Intravitreous tPA + SF6	12.5 μg/0.05 ml + 0.3 ml	20/70	20/16	4
Araújo et al. [42]	2	Intravitreous tPA + SF6	50 μg/0.05 ml + 0.3 ml	20/63	20/20	4
				20/200	20/32	
Hassan et al. [21]	1	Intravitreous tPA + SF6	75 μg/0.15 ml + 0.4 ml	20/200	20/30	9
Holland et al. [49]	2	Intravitreous tPA + SF6 SF6 alone	50 μg + 0.4 ml 0.4 ml	20/125	20/32	12
				20/100	20/32	12
Kung et al. [41]	1	Intravitreal tPA + C3F8	50 μg + 0.3 ml			
Buschini et al. [38]	2	Subretinal tPA	25 μg/0.1 ml	20/100	20/20	3
		Intravitreous tPA + SF6	50 μg + 0.4 ml	20/125	20/63	5
Doi et al. [60]	1	Subretinal tPA	6.9 μg/0.1 ml	HM	20/40	3
Hillenkamp et al. [52]	1	Subretinal tPA + SF6	10–20 μg/0.05–0.1 ml	20/125	20/25	3
Abdul-Salim et al. [81]	1	Intravitreal ranibizumab	0.5 mg	CF	20/63	3

*C3F8* perfluoropropane, *CF* counting fingers, *HM* hand motion, *SF6* sulfurhexafluoride, *tPA* tissue plasminogen activator

### Intravitreal tissue plasminogen activator injection with pneumatic displacement

In 1996 Chen et al. [39] introduced a method involving the intravitreal injection of tPA and gas to displace the blood away from the macula. tPA is a recombinant serine protease which lyses serine residues in plasminogen, activating it into plasmin, enhancing fibrinolysis [40]. The procedure started with dilated fundus exam to check central retinal artery perfusion; then, an anterior chamber paracentesis is realized to prevent an intraocular pressure spike. Finally, an intravitreal injection of tPA is performed, followed by 0.2–0.5 ml of sulfur hexafluoride (SF6) or perfluoropropane (C3F8) gas [28, 38]. After the procedure, the patient should maintain a face down position to support blood displacement [21, 28]. The ideal time interval between injecting intravitreal tPA and starting prone positioning is still debated [21]. The procedure aims to boost enzymatic liquefaction of sub-macular clot with intravitreal tPA, reducing hematic toxic effect, meanwhile long-acting expansile gas generates a pneumatic displacement of the blood, away from the macula [41–43].

How intravitreal tPA can diffuse into subretinal space has not been sufficiently clarified [44, 45]. Actually, Kamei et al. [46] demonstrated that tPA does not diffuse through intact retina in rabbit models. It has been speculated that tPA could pass through retinal micro-lesions and focal breaks in the internal limiting membrane, due to retinal stretching, caused by the hemorrhage itself. In an experimental rabbit model, Coll and colleagues observed liquefaction of a 1-day-old subretinal clot within 24 h after intravitreal TPA injection [44]. Kimura et al. [47] reported complete liquefaction of acute subretinal blood in 6 patients with age-related macular degeneration treated with intravitreal tPA 12–36 h before surgical evacuation of the blood.

Tsuyama et al. [48] published a case report of a 10-year-old male, with traumatic SMH treated with intravitreal tPA injection (12.5 μg in 0.05 ml) followed by 0.3 ml of pure SF6 injection to obtain SMH pneumatic displacement. The procedure was performed with no complications. His baseline VA was 20/70, and improved to 20/16 at 4 month of follow up.

Araújo et al. [42] reported a case series of 6 eyes with SMH, 2 of which due to blunt trauma. All subjects were treated with intravitreal tPA and SF6 achieving a displacement of macular hemorrhage and an improvement of visual acuity.

Hassan et al. [21] treated 15 eyes with acute SMH, one of them was secondary to a blunt trauma. The patient had a 20/200 baseline VA and was treated with 75 μg of intravitreal tPA followed by SF6 injection and 3 day of prone position. Final VA was 20/30 at 9 months of follow-up.

The technique described is cheap and provides a minimum surgical stress to RPE and photoreceptors. Adverse events are rare but could be serious, and include vitreous hemorrhage, hyphema and rebleeding, especially if tPA is injected into fresh hemorrhage, less than 3 days old [49–51]. Remarkably, tPA could provoke retinal toxicity, leading to retinal necrosis and photoreceptors loss. For these reasons, it is recommended to avoid intravitreal dose > 50 μg [21, 39, 52].

### Pneumatic displacement

Due to tPA retinal toxicity, gas displacement alone, followed by face down position, has been proposed for the treatment of traumatic SMH [53]. Gopalakrishan et al. [54] investigated a series of 20 patients with sub macular hemorrhage due to different causes, among which four cases of ocular blunt trauma and a variable visual acuity from hand motion to 20/125. All patients were treated with an intravitreal 0.3-ml injection of pure C3F8 and face down positioning for 5–7 days. Displacement occurred partially or completely in 16 of 20 eyes, and visual acuity remained stable in all eyes during the first year of follow-up. Authors advice to perform this procedure in subjects with symptoms onset less than 30 days.

In a case series of 3 patients with post-traumatic SMH, Balughatta et al. [22] used 0.3 ml of intravitreal pure C3F8, obtaining significant displacement of the SMH and visual recovery in 2 of the 3 cases.

Ohji et al. [55] described a series of five patients treated with 0.4–0.5 ml of pure C3F8 and face down positioning with a complete or partial displacement in all eyes. The authors advise to carry out the treatment within a week of SMH onset, otherwise gas displacement alone could be ineffective.

Holland et al. [49] compared 50 μg of tPA followed by 0.04 ml of pure SF6 injection versus 0.04 ml of pure SF6 injection alone in 2 cases of SMH subsequent to choroidal rupture. VA of the patient treated with the combined procedure improved from 20/125 to 20/63 after 6 days and 20/32 after 10 weeks, stable at 1 year of follow-up. The patient treated with gas alone had a baseline VA of 20/100, improved to 20/32 after 2 months and remained stable at 1 year of follow up. No adverse events were reported in any patient. Both procedures seemed similar in terms of VA improvement, but a combined procedure could result in a faster recovery.

Pneumatic displacement induces an inferior surgical stress compared to intravitreal tPA injection. Interestingly, its efficacy seems to be related to the interval time between SMH onset and treatment. Further investigations are needed to confirm this assumption.

### Subretinal injection of tissue plasminogen activator with pneumatic displacement

The tPA can be injected into subretinal space during a pars plana vitrectomy (PPV), using a 41-gauge needle to release 0.1 ml of tPA, followed by gas tamponade [38, 52, 56].

The technique was first described by Haupert et al. [57] in 2001 who injected tPA directly into subretinal clot using a bent 36-gauge needle. The procedure allows tPA to act with a higher concentration, locally at the site of the hemorrhage, enhancing its fibrinolytic action. At the end of the procedure an intravitreal gas injection aims to displace the blood clot away from the macula. The patient should maintain face down position for at least 24 h [58, 59].

Buschini et al. [38] compared two cases of ocular blunt trauma with SMH development, the first one treated with subretinal injection of tPA (25 μg in 0.1 ml) after PPV and the other one with an intravitreal injection of 50 μg of tPA and 0.4 ml of SF6. The first patient, with 20/100 baseline VA, showed a fast blood reabsorption with an optimal VA outcome (20/20) at 3 months postoperatively. The second patient, with baseline VA of 20/125, showed a persistence of hyperreflective subfoveal fluid on OCT, 1 month after the procedure, with VA of 20/63. At 5 months of follow-up, OCT highlighted focal gaps in photoreceptor layers. Authors speculated that the slower recovery entailed the development of retinal toxicity due to erythrocyte degeneration, compromising the outcome.

In a 13-years-old boy with a traumatic SMH, Doi et al. [60] performed a PPV followed by subretinal tPA (6.9 μg in 0.1 ml) injection. Baseline VA was hand motion. One month after the procedure, due to lens opacity, cataract surgery was executed, in association with intravitreal anti-VEGF injection (0.05 ml bevacizumab), to prevent choroidal neovascularization. At 3 months of follow-up, VA was 20/40 without any other adverse events.

Martel et al. [61] proposed a different therapeutic approach, injecting into subretinal space an association of 0.4 ml of tPA at a concentration of 12.5 μg/0.1 ml (total, 50 μg), 0.1 ml of bevacizumab (2.5 mg), and 0.2 ml of filtered air.

Kung et al. [41] in their retrospective case series, treated 46 consecutive eyes with SMH with 50 μg/0.1 ml of intravitreal tPA and 0.3 ml C3F8. SMH was due to various etiologies such as AMD, PCV, retinal arterial macroaneurysms and ocular trauma (1 case).

In a retrospective, comparative series, Hillenkamp et al. [52] divided their 47 patients in 2 groups, receiving either intravitreal 40 µg of tPA + 20% SF6 (group A) or subretinal 10–20 µg of tPA + 20% SF6 (group B). One of the cases was due to ocular trauma and was included in group B. The authors concluded that treatment B was more effective in terms of hemorrhage displacement and visual recovery but was affected by a higher rate of complications.

The rationale was to maximize hemorrhage displacement within submacular space, acting on friction, gravity and buoyancy, that are the main forces responsible for blood displacement. Friction is reduced by hemorrhage liquefaction due to tPA; upright positioning enhances the gravitational force for inferior SMH displacement; subretinal air bubble decreases SMH buoyancy, allowing a more effective hemorrhage inferior displacement, based on Archimedes’ principle. At the end of the procedure, the vitreous cavity was filled in half with SF6 20%, to keep subretinal air within the macula, and patient should maintain a post-operatory upright position. The procedure may be preferred if SMH does not reach one or both temporal arcade and in case of patients unable to maintain a prone position [61].

Subretinal injection of tPA followed by SF6 tamponade seems to be more effective in terms of visual outcome, when compared to intravitreal tPA injection; nevertheless, it is a more complex surgical maneuver that requires specific surgical skills. Furthermore, it is associated with an increased risk of adverse events, like retinal detachments, cataract development, endophthalmitis, choroidal neovascularization, cystoid macular edema, elevated intraocular pressure or hypotony, pupillary block/angle closure secondary to gas tamponade [52, 62, 63].

### Anti-VEGF agents injections

The anti-vascular endothelial growth factor agents are commonly used to treat neovascular AMD, PCV and proliferative diabetic retinopathy, but recently their therapeutic role in patients with choroidal neovascularization, due to traumatic choroidal rupture, has been analyzed, associated or not with pneumatic displacement [61, 64, 65].

Although there are several papers reporting the use of anti-VEGF agents, alone or in combination of tPA or gas, for the treatment of SMH secondary to AMD [66–74], PCV [75–78] or retinal macroaneurysms [79, 80], in our bibliographic research we found only one report regarding SMH subsequent to ocular trauma.

Abdul-Salim et al. [81] have been the first to present a case report of a 23-year-old male treated with a single dose of 0.5 mg of intravitreal ranibizumab after SMH, secondary to an ocular blunt trauma. VA was counting fingers at 1 m and the dilated fundus examination showed a submacular hemorrhage with an increased central macular thickness (CMT) on OCT. Ranibizumab was injected 20 days after the trauma and VA reached 6/45 at 1 month and 6/18 at 3 months follow-up. OCT showed a CMT reduction and a resolution of the SMH.

Ranibizumab might accelerate the resorption of the hemorrhage with a quick recovery of VA, but the exact mechanism is not fully understood. It has been supposed that anti-VEGF drugs have anti-inflammatory properties, downgrading inflammatory response, besides their anti-angiogenic effect, that reduces vascular permeability. These mechanisms could explain anti-VEGF effectiveness in treatment of SMH, boosting blood’s reabsorption and healing [82–84].

Complications related to intravitreal injection are rare and do not differ from those observed with the use of anti-VEGF drugs for the treatment of other retinal pathologies (i.e. retinal detachment, endophthalmitis, cataract and intraocular pressure elevation) [64].

Anti-VEGF intravitreal injection could be a useful alternative in traumatic SMH treatment, but larger randomized controlled trials are needed to address issues about long-term efficacy and safety.

## Conclusion

**Fig. 2:**
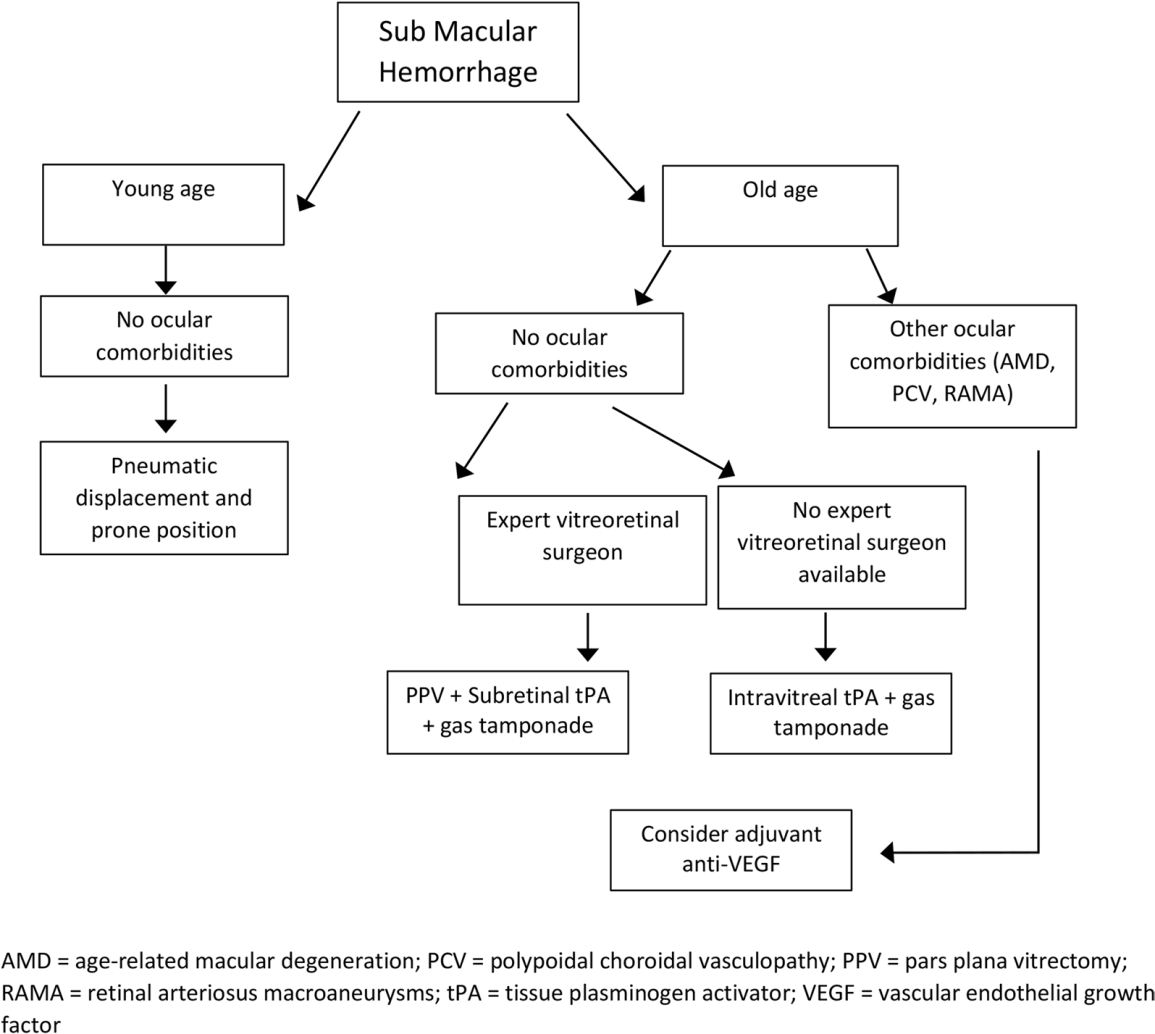
Treatment algorithm in case of traumatic submacular hemorrhage

Traumatic SMH may have an unfavorable evolution if untreated, especially in young patients with sudden vision loss. Nevertheless, management is not clear and depends on various factors such as patient’s age, ocular comorbidities, hemorrhage’s size and thickness and interval time between onset and treatment (Fig. 2). Submacular blood persistence causes progressive photoreceptor toxicity, and for this reason it’s necessary a patient’s thorough evaluation, to analyze preoperative prognostic factors that can determine the final visual outcome.

All procedures obtained consistent results, though the most effective seemed to be PPV, subretinal tPA and 20% SF6 injection, probably due to a quicker liquefaction and displacement of the clot. Limitations concern the greater invasiveness and the higher incidence of complications.

Alternatively, intravitreal injection of tPA and 20% SF6 gas could be a good choice, being less invasive with fewer complications. However, a reduced tPA penetration in the subretinal space, associated with longer blood lasting, could be responsible of lower final VA.

Pneumatic displacement without tPA injection, followed by face down positioning, could be an alternatively treatment in case of young patients without coexisting ocular pathology, being a minimally invasive treatment, with lower risk of complications and morbidity, and a good visual recovery.

Finally, intravitreal anti-VEGF agents have been used successfully to treat submacular hemorrhage due to neovascular AMD, proliferative retinopathy and PCV but few cases, related to ocular trauma, have been described. Results are rising and this treatment could be an alternative or adjuvant therapy to the abovementioned therapy options, however further investigations are warranted to better understand its safety and efficacy.

A limitation of this review was that most of the published literature consists of small studies and case reports, therefore further investigations and larger clinical trials are required to fully understand safety and efficacy of the procedures.

## Data Availability

Not applicable.

## Abbreviations

SMH: sub-macular hemorrhage
tPA: tissue plasminogen activator
VEGF: vascular endothelial growth factor
RPE: retinal pigment epithelium
VA: visual acuity
OCT: optical coherence tomography
SF6: sulfur hexafluoride SF6
C3F8: perfluoropropane
PPV: a pars plana vitrectomy
CMT: central macular thickness
